# Supplementary material for: Antenatal care utilisation and receipt of its components in Nigeria: Assessing disparities between rural and urban areas—A nationwide population-based study
Source: PLoS One. 2024 Jul 15;19(7):e0307316. doi: 10.1371/journal.pone.0307316 (PMC11249263; doi:10.1371/journal.pone.0307316)
Supplement: Attachment — Submitted filename: Antenatal care utilisation in Nigeria-NDHS PLOS one.docx [file pone.0307316.s001.docx]

Antenatal care utilisation in Nigeria: assessing disparities between rural and urban

areas—a nationwide population-based study

Dear Editor,

Thank you for asking me to review this manuscript. I commend the authors for conducting the research and hope that my comments will help strengthen their paper.

Please find my comments below

Summary of the study:

This study investigates antenatal care (ANC) utilization in Nigeria, emphasizing rural-urban disparities. Using 2018 Nigeria Demographic and Health Survey data, ANC utilization, component receipt, and associated factors were analyzed. Results reveal significant disparities, with higher ANC use in urban areas. Factors such as education, health insurance, and maternal autonomy influence ANC utilization nationally, with variations between rural and urban contexts. Disparities persist, particularly affecting rural residents and socioeconomically disadvantaged mothers.

Overall impression

The paper will benefit from minor revisions. See comments below.

Abstract

1. Well-written

Consider a more actionable recommendation rather than repeating the phrase ‘vulnerable populations’

Introduction

2. The introduction could benefit from a more focused discussion on the specific research objectives and the rationale for examining rural-urban disparities in ANC utilization.
3. While it briefly mentions the previous study by the authors and the use of data disaggregation, a clearer explanation of why rural-urban disparities is particularly important in the Nigerian context would enhance the reader's understanding. Additionally, how the previous study differs from this except in the use of different datasets (ie 2013 vs. 2018).

Methods

4. Information on the study area, and contextual factors affecting ANC use is needed for a non-Nigerian reader to understand this. Consider shortening the data source to incorporate this.
5. We access the current data 158 for research purpose in June – August 2023. Should this be ‘accessed’?
6. Please clarify the role of the Children Recode dataset since it is focused on the record for children born in the preceding past 5 years. If this was used to access the mother’s information then make this explicit.
7. More details on the model building and comparison and the variables. What was used to compare models such as the Hosmer and Lemeshow tests and the area under the receiver operating characteristic curve (AUC).
8. Any need for sensitivity analysis? Why and why not
9. How did you choose variables to include the multivariable binary logistic regression analyses
10. Any information on cluster, strata
11. A DAG would be useful for this research and for model building as well as identifying variable types eg. Confounders, instrumental variables. Independent predictors, Mediators etc This will help decide what goes into the model and as what.
12. How was missingness handled eg. multiple imputation

Discussion and conclusion

13. It will be good to discuss using terms and concepts like intersectionality and social determinants of health which best describe the factors affecting ANC use
14. The discussion could benefit from a more nuanced focus on the implications for maternal and neonatal health outcomes in Nigeria
15. Consider making the conclusion shorter and removing proportions, including specific strategies for addressing the identified gaps. The below is by no means acceptable and quite non-specific!

‘Efforts to bridge the rural-urban, address regional disparities, and prioritise vulnerable populations are imperative for enhancing ANC utilisation and maternal and perinatal health outcomes in Nigeria.’
