# Supplementary material for: Antenatal care utilisation and receipt of its components in Nigeria: Assessing disparities between rural and urban areas—A nationwide population-based study
Source: PLoS One. 2024 Jul 15;19(7):e0307316. doi: 10.1371/journal.pone.0307316 (PMC11249263; doi:10.1371/journal.pone.0307316)
Supplement: Attachment — Submitted filename: Response to reviewers.docx [file pone.0307316.s002.docx]

**Authors’ response to reviewers’ comments**

Title: Antenatal care utilisation in Nigeria: assessing disparities between rural and urban areas—a nationwide population-based study (PONE-D-24-05470)

Authors:

Emmanuel O Adewuyi, Asa Auta, Mary I Adewuyi, Aaron Akpu Philip, Victory Olutuase, Yun Zhao, Vishnu Khanal

Dear Editor,

We appreciate the meticulous review of our manuscript and your insightful comments, as well as those of the reviewers. We have thoroughly considered the comments and revised the manuscript accordingly. Our point-by-point responses are highlighted in blue colour below. Line number references here are with regards to the corrected ‘clean’ (unmarked) manuscript version.

In addition to the comments and recommendations of the reviewers:

1. We made a minor correction to the title to effectively reflect the contents of the study and maximise its potential impacts.

New title:

**Antenatal care utilisation and receipt of its components in Nigeria: assessing disparities between rural and urban areas—a nationwide population-based study**

2. We made a few corrections here and there to improve our manuscript's quality and readability.
3. We proofread and spell-checked our manuscript.

**Editorial comments:**

1. Please ensure that your manuscript meets PLOS ONE's style requirements.

Response: We have checked and endeavour to ensure our manuscript conforms to the Plos One style

2. Please include your full ethics statement in the ‘Methods’ section of your manuscript file.

Response: We have included an ethics statement in the Methods section as follows:

*‘The Nigerian National Health Research Ethics Committee granted ethics approval for the survey. Survey participants who were 18 years or older provided informed written consent, and consent was obtained from their parents or guardians if they were below 18. We received approved access to use the data, and given our research is based on a secondary data analysis, no further ethical clearance was required to conduct this study. Access to the data used in this study is freely available online (https://dhsprogram.com/data/available-datasets.cfm) after obtaining the necessary approval from the Demographic and Health Survey (DHS) program’ (lines 204 – 211).*

3. Please upload a copy of Supporting Information Figure/Table/etc. Supporting information which you refer to in your text on page 24 and 25.

Response: We upload supporting information listed in the referenced pages as follows:

- All Tables were included within the manuscript
- All Figures were uploaded at submission point as required.

4. Please review your reference list to ensure that it is complete and correct. If you have cited papers that have been retracted, please include the rationale for doing so in the manuscript text, or remove these references and replace them with relevant current references. Any changes to the reference list should be mentioned in the rebuttal letter that accompanies your revised manuscript.

Response: We confirm that we have checked our reference list and they are correct. We did not cite retracted papers (to the best of our knowledge). Due to the revision requested by the reviewers, the number of references in our manuscript increased from 41 to 56. We confirm that all included references are relevant and appropriate.

**Editor Comments**

1. Two experts have reviewed the paper noting some limitations in reporting, that might require some changes in the analysis depending on the answer to the questions. Please pay attention, in particular, to the detailed suggestions of reviewer 2.

Response:

We thank the editor and reviewers for their feedback on our manuscript. We have carefully considered (and paid close attention to) all the comments and responded accordingly, making necessary corrections or providing rebuttals as appropriate.

2. In particular, remember PLOS ONE policy regarding replication studies, that is of concern given the similarity to your previous study: If a submitted study replicates or is very similar to previous work, authors must provide a sound scientific rationale for the submitted work and clearly reference and discuss the existing literature.

Response:

We appreciate the editor's comment. We confirm no overlap between the present study and our previous publication (PMID: 29782511)[1]. Also, we did not utilise any information from the previous work except where appropriately cited. Below, we outline some differences between the two studies:

- Data used: Our previous publication (PMID: 29782511)[1] was based on data from the Nigeria Demographic and Health Survey (NDHS) 2013, while the current manuscript utilised entirely new and more recent data from the 2018 NDHS (the most current in the series of such surveys in Nigeria). Therefore, the present manuscript provides updated and more relevant evidence.
- Coverage: The previous publication solely addressed ANC underuse. Conversely, the current manuscript extends its coverage to include ANC utilisation and the quality of ANC received, thereby offering a more comprehensive assessment. The quality of ANC has been an issue and has rarely been prioritised in the literature from low-middle-income countries. Our study assessed ANC components and provided insights into differences across national, rural and urban settings in Nigeria.
- Definition: The previous publication adhered to the Focused ANC model, which typically requires a minimum of four ANC contacts. However, the current manuscript adopts the new WHO recommendation of eight or more contacts [2, 3], reflecting alignment with updated global health guidelines.

(Web link to reports on WHO's new guideline on ANC:

<https://www.who.int/news/item/07-11-2016-new-guidelines-on-antenatal-care-for-a-positive-pregnancy-experience>)

Thus, the current study builds upon previous research by utilising more recent data and expanding the scope of analysis to include both ANC utilisation and quality. The adoption of updated WHO guidelines underscores the relevance and rigour of our findings in informing maternal healthcare policies and practices in Nigeria with a focus on rural-urban differences. We have updated our manuscript to reflect the differences as appropriate.

**Reviewers' comments**

**Reviewer #1:**

The main concept is fine. Data and research methodlogy including with statistics is acceptable. However, transition between pargraph and table is not well written. It needs to check and revise a bit. Besides, discussion needs to related to their ressults. I hope they able to change and revise.

Response:

We thank the reviewer for the insightful, helpful, and constructive feedback. We have carried out a thorough revision, improving on transition between paragraphs and the presentation of our Tables. Also, we have improved or even rewritten some parts of the discussion, ensuring it focuses on findings.

Specifically, we have made the following revisions, which can be seen in the tracked version of our revised manuscript:

- We improved the readability and presentation of the abstract section, ensuring the recommendations are more actionable.
- We improved the presentation and flow of the introduction section, ensuring that transitions between paragraphs are seamless and connect well.
- We improved the presentation of our Tables to make them compact and easy to read. Our tables are relatively large but have been presented in ways that make them easy to follow.
- We carried out a thorough proofreading of our manuscript and made corrections as necessary.

**Reviewer #2:**

Overall impression: The paper will benefit from minor revisions.

**Abstract**

1. Well-written. Consider a more actionable recommendation rather than repeating the phrase ‘vulnerable populations’

Response:

We appreciate the reviewer for the insightful, helpful, and constructive feedback.

We have revised the abstract section for clarity. Importantly, we provide more actionable recommendations (with consideration for word count limit) and avoid repetition of words or phrases, including ‘vulnerable populations’.

The conclusion (with brief recommendations) in the abstract section now reads:

*‘Our study reveals significant disparities in ANC utilisation and components across Nigeria, with rural residents, particularly in northern regions, socioeconomically disadvantaged and teenage mothers facing notable challenges. A multifaceted approach prioritising the interplay of intersectional factors like geography, socioeconomic status, education, religion, ethnicity, and gender dynamics is essential. Key strategies should include targeted interventions to promote educational opportunities, expand health insurance coverage, leverage internet and context-specific media, and foster socioeconomic empowerment, prioritising underserved populations’ (lines 57 – 63).*

**Introduction**

2. The introduction could benefit from a more focused discussion on the specific research objectives and the rationale for examining rural-urban disparities in ANC utilization.

Response:

We appreciate the merit of this comment and have rewritten several aspects of the introduction section. Specifically, we made our introduction more focused and highlighted specific research objectives, backed by the rationale for focusing on rural-urban disparities in ANC utilisation. Below, we provide some excerpts:

- Specific objectives:

*‘The present study's objectives, thus, are to assess ANC utilisation of eight or more contacts (following the new WHO guidelines) and evaluate its variation across national, rural, and urban settings in Nigeria. Secondly, we aim to examine the receipt of ANC components and their differences across these settings. Additionally, we investigate geographic, demographic, socioeconomic, and healthcare-related factors associated with ANC use and assess how they differ across national, rural, and urban settings’ (lines 149 – 155).*

- Rationale for rural-urban assessment:

*‘However, several available studies are limited by their generalised approach, primarily focusing on national estimates using pooled datasets, which may inadvertently mask differences between and within population groups [1]. There is a growing appreciation of the importance of using high-quality, disaggregated data studies as an evidence-based approach to address access, survival, and equity disparities across socioeconomic and geographic divides [4-6]. This type of data disaggregation aligns with the WHO’s framework for monitoring progress towards Universal Health Coverage, which emphasises the need to disaggregate all socioeconomic and demographic strata measures to better assess equity in intervention coverage, among other factors [7]’ (lines 115 – 122)*

3. While it briefly mentions the previous study by the authors and the use of data disaggregation, a clearer explanation of why rural-urban disparities is particularly important in the Nigerian context would enhance the reader's understanding. Additionally, how the previous study differs from this except in the use of different datasets (ie 2013 vs. 2018).

Response: We have provided additional information and refined our approach in the introduction to clarify our study’s focus on data disaggregation by the rural-urban divide in Nigeria. Also, we highlight how the present study differs from our previous publication in this area.

- Data disaggregation approach:

*‘Importantly, in its newly released 2024 ‘Operational Framework for Monitoring Social Determinants of Health Equity’ [8], the WHO emphasises that data disaggregation is crucial for monitoring social determinants of health as it enables the identification and analysis of health inequities across different population subgroups. For example, by breaking down data by factors such as geographic location (rural-urban), demographic (age, gender), socioeconomic, and ethnicity, it is possible to uncover disparities and target interventions more effectively [8]. This detailed analysis approach supports the goal of the 2030 Agenda for Sustainable Development to ‘leave no one behind’ by tracking progress across various Sustainable Development Goals (SDGs) and ensuring equitable access to health-promoting resources and services [8].*

*Specifically, Nigeria's diverse demographics and healthcare infrastructure underscore the need for understanding ANC utilisation across various subpopulations [9]. The country's notable geographic, demographic and socioeconomic disparities can exacerbate existing health inequities and have implications for policymaking and resource allocation efforts to improve maternal healthcare services. Identifying areas where resources and interventions are most needed can, for example, enable tailored strategies to bridge the gap in ANC utilisation and improve maternal and neonatal health outcomes nationwide’ (lines 122 – 137).*

- Comparison with our previous publication:

*‘Consistent with this premise, our previous study used data disaggregation to gain insight into Nigeria's underutilisation of ANC services, focusing on differences between rural and urban residences [1]. Given the importance of this subject and the new WHO recommendations, we aim to build upon the study by investigating ANC service utilisation and quality (i.e., receipt of ANC components) in Nigeria using the latest nationally representative demographic and health survey data [9].*

*While our prior study [1] adhered to the focused ANC model, requiring a minimum of four ANC contacts, the current study adopts the new WHO recommendation of eight or more contacts [2, 3, 10], reflecting alignment with the updated global health guidelines. In addition, we expanded our investigation to examine disparities in receiving essential ANC components between rural and urban areas following the recommended data disaggregation approach. These components, including iron supplementation, tetanus vaccinations, medicines for intestinal parasites, blood pressure checks, and various laboratory tests [2, 3], are crucial for promoting maternal and neonatal health’ (lines 137 – 149).*

**Methods**

4. Information on the study area, and contextual factors affecting ANC use is needed for a non-Nigerian reader to understand this. Consider shortening the data source to incorporate this.

Response: We have provided a brief sub-section for the study setting, introducing the country and setting the tone for understanding our study. This sub-section reads:

*‘Nigeria is Africa's most populous country, with over 220 million people, nearly half (46.48%) of whom reside in rural areas. The country comprises over 374 ethnic groups and languages, organised into 36 states and the Federal Capital Territory, with six geopolitical zones (regions). Further administrative subdivisions include local government areas (LGAs) within states and wards, also known as enumeration areas (EAs), within LGAs. Approximately 63% of Nigerians live in multidimensional poverty [11], highlighting socioeconomic disparity. Healthcare provision involves both public and private sectors, with public health services shared among the three tiers of government: primary (LGAs), secondary (states), and tertiary level of care (federal). In 2017, a notable shift occurred in ANC practices following the adoption of the 2016 WHO ANC guideline [2, 3], increasing ANC contacts from a minimum of four to eight in Nigeria [10]. Nigeria struggles with poor population health outcomes. However, national statistics can mask the profound disparities across geographic and socioeconomic divides [12], hence, the rural-urban data disaggregation approach used in the current study’ (lines 168 – 179).*

5. We access the current data 158 for research purpose in June – August 2023. Should this be ‘accessed’?

Response: Thank you for picking the grammar error; we have corrected it to ‘accessed’ (line 201).

6. Please clarify the role of the Children Recode dataset since it is focused on the record for children born in the preceding past 5 years. If this was used to access the mother’s information then make this explicit.

Response: We have provided the information requested, which reads:

*‘The KR dataset contains individual records for each child born to the interviewed women within the five years before the survey. It provides extensive details regarding the child's pregnancy, postnatal care, immunisation, general health status, and corresponding data for their mothers. We utilise the data available in this dataset to access the mother's information. We received approved access to use the data, and no further ethical clearance was required to conduct this study’ (lines 202 – 206).*

*Link to the DHS website for more information:* [*https://dhsprogram.com/data/Dataset-Types.cfm*](https://dhsprogram.com/data/Dataset-Types.cfm)

7. More details on the model building and comparison and the variables. What was used to compare models such as the Hosmer and Lemeshow tests and the area under the receiver operating characteristic curve (AUC).

Response: the survey producing the data for our analysis is based on a complex sample design with survey weights, clustering, and stratification, which we reckon can complicate model fit testing in logistic regression analysis. We did not use Hosmer-Lemeshow or AUC tests in the present study. The Hosmer-Lemeshow test is one of the most widely applied tests in practical applications, assuming a simple random sample. However, this assumption may not be valid for survey data based on complex sample designs. Notably, the test is unavailable in the CS of SPSS (to our knowledge). Given the nature of our study and to avoid complexities, we followed approaches in previous studies where these tests were not used [1, 13-20]. To address concerns about likely statistical errors, we note as follows:

*‘To minimise potential statistical errors, we meticulously double-checked our analysis process to ensure all variables meeting the inclusion criteria were included in our models. Additionally, we rigorously validated the effectiveness of our backward elimination modelling by testing the final parsimonious models against potential confounding variables and factors previously reported to be associated with ANC’ (lines 265 – 269).*

8. Any need for sensitivity analysis? Why and why not

Response: Sensitivity analysis in logistic regression for survey data with stratified sampling design may be complicated, with the potential for biased results. While we cannot rule it out, to the best of our knowledge, most previous studies in this area of research do not perform sensitivity analysis [1, 13-20].

9. How did you choose variables to include the multivariable binary logistic regression analyses

Response: we provided information on our multivariable model building, which reads as follows:

*‘In our model-building process, we only included variables that showed statistical significance (p < 0.05 in the Chi-Square test) in the initial model of the multivariable regression analyses’ (lines 261 – 263).*

So, variables significantly associated with ANC use at p < 0.05 (in the univariate analysis) were selected for inclusion in our multivariable logistic regression modelling.

10. Any information on cluster, strata

Response: Yes. We noted in our manuscript as follows:

*‘The NDHS 2018, the sixth edition in its series, used a two-stage stratified cluster sampling method involving about 42,000 households and 1,400 clusters [9]’ (lines 189 – 190)’.*

*A*lso, we mentioned in the statistical analysis sub-section that:

*‘In all analyses, we used the Complex Sample analysis method, considering the stratified design and the sampling weight of the 2018 NDHS’.*

In our revised manuscript, we elaborate more on this information for clarity as follows:

*‘In all analyses, we utilised SPSS's complex sample function to adjust for the sample weight and the multistage cluster design of the NDHS 2018 data. This approach addresses the complexities of cluster sampling and stratification, ensuring the statistical reliability of our estimates by accounting for the unequal selection probabilities inherent in the survey design. To facilitate this adjustment, we developed a complex sample plan outlining the necessary considerations for SPSS during data analysis, including sampling weights, strata, and clusters (lines 275 – 281)’.*

11. A DAG would be useful for this research and for model building as well as identifying variable types eg. Confounders, instrumental variables. Independent predictors, Mediators etc This will help decide what goes into the model and as what.

Response: We appreciate the reviewer's suggestion. However, we did not use DAG in the present study. We followed well-established methods in selecting variables for inclusion in the model-building process in our study, as more comprehensively described in our methods section. For example, we stated as follows:

*‘We adopted Andersen's behavioural model [21] as the conceptual framework for selecting explanatory variables in this study, which is consistent with practice in previous studies [1, 14, 22]. Briefly, Andersen's behavioural model is widely used as a framework in healthcare research, especially in studies examining factors influencing health service utilisation. In the context of ANC utilisation, the model considers three key components: predisposing factors (e.g., demographics), enabling factors (e.g., access to resources), and need factors (e.g., perceived health needs) [21]. Considering variables captured in the NDHS 2018 [9], we apply this model to assess how a range of factors are associated with a woman's decision to seek ANC services. The model, as used in our study, is depicted in Fig 1. Following the example of previous studies [1, 22], we reviewed and carefully selected a total of twenty-two explanatory variables for inclusion in our analysis. We broadly grouped these variables into four categories: external environmental, predisposing, enabling, and need factors (Fig 1)’ [lines 228 – 238].*

12. How was missingness handled eg. multiple imputation

Response: we noted as follows:

‘*Mothers who responded with 'don't know' or had missing information regarding the question "How many times did you receive antenatal care during this pregnancy?" were excluded from our analyses.’*

To address the reviewer’s question fully, we also added:

*‘We excluded missing data or responses recorded as ‘don’t know’ in all analyses’ (lines 270 – 271).*

**Discussion and conclusion**

13. It will be good to discuss using terms and concepts like intersectionality and social determinants of health which best describe the factors affecting ANC use

Response: We thank the reviewer for this comment. We made extensive corrections and rewrote substantial parts of our discussion section. We have now followed the intersectionality and social determinants of health approach in discussing factors associated with ANC. The opening paragraph of this approach:

*‘**Following multivariable analyses, we identified several factors associated with ANC utilisation in Nigeria across rural and urban areas, revealing a complex interplay of intersectional dynamics. Often rooted in systemic inequalities, these dynamics can be better comprehended through the lens of social determinants of health [23-25]. The dynamics encompass various elements, including geographic location, regional disparities, healthcare system functionality, individual characteristics, socioeconomic status, and cultural influences, all intricately shaping access to and utilisation of healthcare services. Briefly, social determinants of health encapsulate how individuals live, learn, work, and play, shaped by the distribution of money, power, and resources [25]. These determinants profoundly influence a diverse spectrum of health outcomes and quality-of-life indicators [24]. Conversely, intersectionality elucidates how various systems of marginalisation and privilege intersect, intertwining to form unique experiences for individuals and communities [23]. This concept offers a framework for comprehending lived realities without reducing individuals to singular characteristics [23]. Therefore, promoting maternal and neonatal health outcomes in Nigeria necessitates a holistic approach that acknowledges and addresses the intertwined effects of social determinants and intersectional factors contributing to health inequities in the country. By embracing this comprehensive perspective, policymakers and healthcare stakeholders can devise more effective strategies to dismantle barriers to ANC use and advance the health and well-being of individuals and communities’ (lines 458 – 475)*.

We thoroughly discussed our findings on factors associated with ANC in light of this concept (lines 476 – 595).

14. The discussion could benefit from a more nuanced focus on the implications for maternal and neonatal health outcomes in Nigeria.

Response: We thank the reviewer for this comment. We have further improved and focused on the implications of our study for maternal and neonatal health outcomes in Nigeria (discussion section).

15. Consider making the conclusion shorter and removing proportions, including specific strategies for addressing the identified gaps. The below is by no means acceptable and quite non-specific!

‘Efforts to bridge the rural-urban, address regional disparities, and prioritise vulnerable populations are imperative for enhancing ANC utilisation and maternal and perinatal health outcomes in Nigeria.’

Response: We have shortened the conclusion section and removed ‘proportions’ as requested. We improved on the presentation of our recommendation and removed the sentence referenced by the reviewer. The conclusion now reads:

*‘Our study reveals substantial disparities in ANC use, its components, and associated factors across Nigeria's national, rural, and urban settings. Nationally, ANC use is low, with only one-fifth of mothers achieving the recommended ≥8 contacts. Notable rural-urban disparities exist, with approximately 3.5 times higher ANC use in urban areas. The North-East region exhibits disproportionately lower ANC utilisation nationally and in urban settings, while the North-West shows the lowest use in rural areas. Our findings also highlight a concerning decline in receiving ANC components, such as iron supplementation, which particularly affects rural residents. Maternal and husband education levels, health insurance coverage, and maternal autonomy are significant predictors of ANC use across Nigeria. All ethnic groups showed higher ANC odds than the Hausa/Fulanis in urban areas, while only the Yorubas had greater odds in rural areas. Nationally, internet use was significant, with television being significant in urban areas and factors like maternal working status, wealth, birth type, religion, and radio listenership significant in rural settings.*

*Addressing the disparities observed in this study requires an intersectional approach that considers how multiple social determinants and identities, such as socioeconomic status and geographic location, intersect to impact healthcare access and utilisation. Tackling deep-seated social inequalities and mitigating barriers rooted in diverse cultural practices are essential for promoting culturally sensitive ANC services. Universal health insurance can minimise financial barriers, and enhancing internet access can facilitate information dissemination, health promotion, and telemedicine. Targeted media channels like radio and television can educate and disseminate information to marginalised communities. Empowering individuals and communities through educational opportunities can foster socioeconomic empowerment, maternal autonomy, and improved ANC use’ (lines 620 – 644).*

**References**

1.	Adewuyi, E.O., et al., *Prevalence and factors associated with underutilization of antenatal care services in Nigeria: A comparative study of rural and urban residences based on the 2013 Nigeria demographic and health survey.* PLOS ONE, 2018. **13**(5): p. e0197324.

2.	World Health Organization, *WHO recommendations on antenatal care for a positive pregnancy experience*. 2016: World Health Organization.

3.	World Health Organization, *WHO recommendations on antenatal care for a positive pregnancy experience: summary: highlights and key messages from the World Health Organization's 2016 global recommendations for routine antenatal care*. 2018, World Health Organization.

4.	Hosseinpoor, A.R., et al., *Equity-oriented monitoring in the context of universal health coverage.* PLoS Med, 2014. **11**(9): p. e1001727.

5.	UNICEF, *Committing to Child Survival: A Promise Renewed, Progress Report 2014. 2014.* New York, USA: UNICEF, 2015.

6.	Kauh, T.J., J.n.G. Read, and A. Scheitler, *The critical role of racial/ethnic data disaggregation for health equity.* Population research and policy review, 2021. **40**(1): p. 1-7.

7.	World Health Organisation and World Bank Group, *Monitoring progress towards universal health coverage at country and global levels: framework, measures and targets*. 2014 Geneva: WHO

8.	World Health Organization, *Operational framework for monitoring social determinants of health equity.* 2024: Geneva.

9.	Nigeria Population Commission, *Nigeria demographic and health survey 2018*. 2019, NPC, ICF.

10.	Federal Ministry of Health Nigeria, *Antenatal Care: An Orientation Package for Health Care Providers*. 2017.

11.	National Bureau of Statistics, *Nigeria multidimensional poverty index.* 2022: Abuja.

12.	Abubakar, I., et al., *The Lancet Nigeria Commission: investing in health and the future of the nation.* The Lancet, 2022. **399**(10330): p. 1155-1200.

13.	El-Khatib, Z., et al., *Patterns and Predictors of Insufficient Antenatal Care Utilization in Nigeria over a Decade: A Pooled Data Analysis Using Demographic and Health Surveys.* International Journal of Environmental Research and Public Health, 2020. **17**(21): p. 8261.

14.	Envuladu, E.A., et al., *Differential Associated Factors for Inadequate Receipt of Components and Non-Use of Antenatal Care Services among Adolescent, Young, and Older Women in Nigeria.* International Journal of Environmental Research and Public Health, 2023. **20**(5): p. 4092.

15.	Agho, K.E., et al., *Factors associated with inadequate receipt of components and use of antenatal care services in Nigeria: a population-based study.* International health, 2018. **10**(3): p. 172-181.

16.	Fagbamigbe, A.F. and E.S. Idemudia, *Barriers to antenatal care use in Nigeria: evidences from non-users and implications for maternal health programming.* BMC pregnancy and childbirth, 2015. **15**: p. 1-10.

17.	Ekholuenetale, M., F.O. Benebo, and A.F. Idebolo, *Individual-, household-, and community-level factors associated with eight or more antenatal care contacts in Nigeria: Evidence from Demographic and Health Survey.* Plos one, 2020. **15**(9): p. e0239855.

18.	Alex-Ojei, C.A. and C.O. Odimegwu, *Correlates of antenatal care usage among adolescent mothers in Nigeria: a pooled data analysis.* Women & Health, 2021. **61**(1): p. 38-49.

19.	Bolarinwa, O.A., et al. *Spatial patterns and multilevel analysis of factors associated with antenatal care visits in Nigeria: Insight from the 2018 Nigeria Demographic Health Survey*. in *Healthcare*. 2021. MDPI.

20.	Nwosu, C.O. and J.E. Ataguba, *Socioeconomic inequalities in maternal health service utilisation: a case of antenatal care in Nigeria using a decomposition approach.* BMC public health, 2019. **19**(1): p. 1-11.

21.	Andersen, R.M., *Revisiting the behavioral model and access to medical care: does it matter?* Journal of health and social behavior, 1995: p. 1-10.

22.	Adewuyi, E.O., et al., *Prevalence and factors associated with non-utilization of healthcare facility for childbirth in rural and urban Nigeria: Analysis of a national population-based survey.* Scandinavian Journal of public health, 2017. **45**(6): p. 675-682.

23.	Hoang, T.-M.H. and A. Wong, *Exploring the Application of Intersectionality as a Path toward Equity in Perinatal Health: A Scoping Review.* International Journal of Environmental Research and Public Health, 2023. **20**(1): p. 685.

24.	Macias-Konstantopoulos, W.L., et al., *Race, Healthcare, and Health Disparities: A Critical Review and Recommendations for Advancing Health Equity.* West J Emerg Med, 2023. **24**(5): p. 906-918.

25.	Marmot, M., *Social determinants of health inequalities.* The lancet, 2005. **365**(9464): p. 1099-1104.
